# Dysbiosis in intestinal microbiome linked to fecal blood determined by direct hybridization

**DOI:** 10.1007/s13205-020-02351-w

**Published:** 2020-07-28

**Authors:** Concetta Cafiero, Agnese Re, Salvatore Pisconti, Marina Trombetti, Mariarita Perri, Manuela Colosimo, Gerardo D’Amato, Luca Gallelli, Roberto Cannataro, Clelia Molinario, Alessia Fazio, Maria Cristina Caroleo, Erika Cione

**Affiliations:** 1 Oncology Unit, SG Moscati Hospital of Taranto, Taranto, Italy; 2 Laboratory of Medical Genetics, Alessandria Artemisia, Rome, Italy; 3 CNR-Institute of Cell Biology and Neurobiology, Rome, Italy; 4 Dietetics and Aesthetic Medicine Section, Alessandria Artemisia, Rome, Italy; 5 Department of Pharmacy, Health and Nutritional Sciences-Department of Excellence 2018-2022, University of Calabria, 87036 Rende, CS Italy; 6 Department of Microbiology and Virology, Pugliese Ciaccio Hospital, Catanzaro, Italy; 7 Department of Endocrine and Metabolic Surgery, Policlinico Universitario A Gemelli-Università Cattolica del Sacro Cuore, Rome, Italy; 8 Department of Endocrine and Metabolic Surgery, Mater Olbia Hospital, Olbia, Italy; 9 Clinical Pharmacology and Pharmacovigilance Unit, Department of Health Sciences, Mater Domini Hospital, University of Catanzaro, Catanzaro, Italy; 10 Nutrics, Nutritional Center, Luzzi, CS Italy

**Keywords:** Direct detection, Microbiome, Proteobacteria, *Clostridium difficile*, Eubiosis, Dysbiosis

## Abstract

The important physiological and pathophysiological roles of intestinal human microbiome (HMB) in human health have been emerging, owing to the access to molecular biology techniques. Herein we evaluated, for the first time, the intestinal HMB through direct hybridization approach using n-counter flex DX technology which bypasses the amplification procedure currently applied by other technologies to study the human microbiome. To this purpose, a clinical study was carried out on fecal samples, recruiting both healthy volunteers (N-FOB) and subjects positive for occult blood (P-FOB). A relevant custom panel of 79 16S rRNA target gene was engineered and 32 of them displayed a variation between the two clusters of subjects. Our findings revealed that bacteria belonging to Proteobacteria have higher distribution in P-FOB describing dysbiosis. Similarly, Bacteroidetes and Firmicutes phylum display high distribution in P-FOB. Of interest, the presence of *Clostridium difficile* that belongs to Firmicutes phylum displayed about 70% of low presence in N-FOB compared to P-FOB subjects. Only one bacterium belonging to the Actinobacteria phylum, the *Bifidobacterium bifidum*, was present.

**Electronic supplementary material:**

The online version of this article (10.1007/s13205-020-02351-w) contains supplementary material, which is available to authorized users.

## Introduction

Around 10^^12^ (trillion) complex microbial communities composed of fungi, yeasts, viruses and bacteria reside in the digestive tract, which constitute the human microbiome (HMB) (Marchesi et al. 2016). Its metabolism as well as its genetic set interacts with the host organism defining a close symbiotic relationship (Fischbach and Segre 2016). As a result, the bacterial composition mirrors the sophisticated commensality interplay that is established with the host organism and within the microbial community (Thursby and Juge 2017; Khangwal and Shukla 2019). Intestinal HMB changes with aging and metabolic disorder, and may contribute to the decline of nutrients’ absorption (Dahiya et al. 2017; Kastl et al. 2020). It has been shown that by proper nutritional intervention, HMB can be restored and balanced sustaining eubiosis (Nagpal et al. 2018; Salazar et al. 2017; Wu and Wu 2012). Eubiosis is also re-established by antiviral therapy in persistent hepatitis B virus (HBV) infection mouse model (Li et al. 2020). HMB is capable of guaranteeing the well-being of the entire organism and its role is essential for the immune system of the host organism (Wu and Wu 2012; Mu et al. 2016). Environmental factors, poor lifestyles, psycho-physical stress, overnutrition, and pharmacological treatments are able to modify HMB, defining the dysbiosis (Karl et al. 2018). This latter condition is often linked to the lack of intestinal homeostasis which in turn correlates to a wide range of inflammatory conditions (Wen and Duffy 2017). Current knowledge concerning intestinal HMB, using test based on 16S rRNA gene target, points out to the existence of a community of almost 1000 bacterial species classified into five phyla: *Actinobacteria*, *Bacteroidetes*, *Firmicutes*, *Proteobacteria* and *Verrucomicrobia* (Rajilic-Stojanovic M and de Vos WM, 2014). The results achieved thus far via high-throughput sequencing (HTS) platforms are very interesting, but the workflow, which includes the library preparation protocols and the enzymatic amplification of the nucleic acid, could lead to different results among the HTS platform used (Salipante et al. 2016; D’Amore et al. 2016; Loman et al. 2012; Lam 2011; Quail et al. 2012; Clooney et al. 2016; Mohammadi et al. 2019). Besides that, sample collection and bacterial DNA extraction as well as the 16S rRNA gene target region represent other important points in the assessment of intestinal HMB (Pollock et al. 2018; Rintala et al. 2017). Therefore, concerns about using HMB analysis is still debated, limiting it to routinely clinical practice (Pollock et al. 2018). In this framework, the assessment of the intestinal HMB in the medical setting may be helpful to dissect symptoms such as episodic colitis attack, diarrhea, constipation, flatulence, and intestinal discomfort (Chichlowski and Rudolph 2015; Simrén et al. 2013). Moreover, intestinal HMB analysis can be fundamental to develop therapeutic and personalized nutritional interventions (Seo et al. 2013). In this work, we attempted to overcome these concerns. To minimize experimental variation, fecal samples were collected and stored with OMNI gene GUT, which proved as a reliable and convenient system to study intestinal HMB (Panek et al. 2018). We performed bacterial DNA extraction with automatic system MagCore HF16 Plus with some modification to improve DNA yield; to avoid library preparation, we performed direct hybridization with n-counter flex DX. To evaluate dysbiosis, fecal occult blood (FOB) samples were collected from patients enrolled via a clinical trial. Of note, several inflammatory intestinal disorders are related to FOB which is not strictly related to colon cancer (Walker 1990; Libby et al. 2018). In this scenario, we have designed in July 2017 the first custom panel “CDR_CNV_Bc_miCrobioTA22586” with 79 bacteria 16S rRNA target genes representative of gut health and impaired in gut inflammation status (Tarallo et al. 2019). *Alicyclobacillus acidophilus*, *Rhizobium radiobacter* and *Salinibacter ruber* are not present in the human gut and were used as negative control of human microbiota bacteria 16S rRNA gene. ACTB, GAPDH and HDAC3 genes were used for monitoring human DNA contamination.

## Materials and methods

### Study design, sample collection and HMB community

**Fig. 1:**
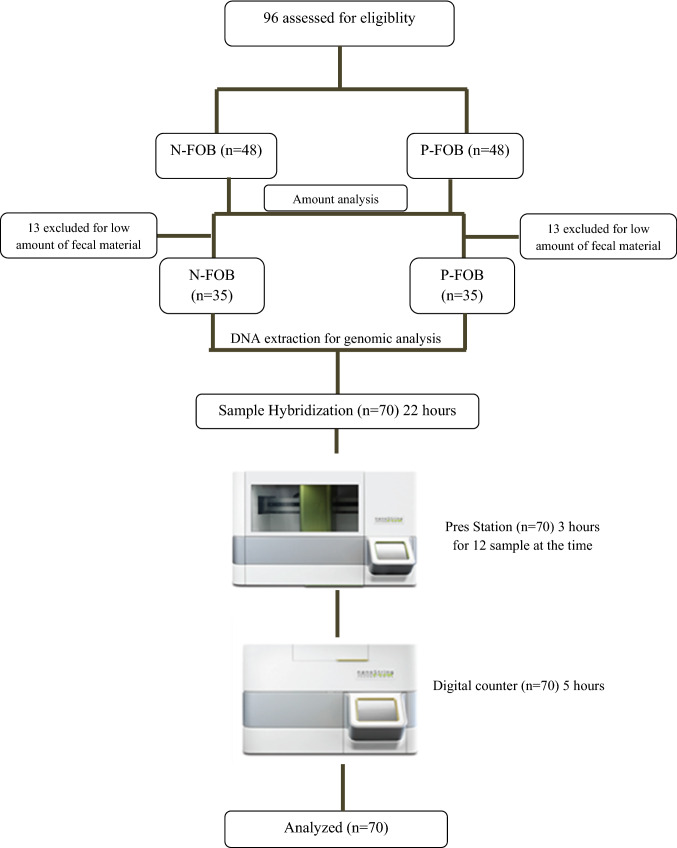
Study design and analytical workflow. Initially, 48 samples for each group, N-FOB, and P-FOB were collected, 13 samples for each group resulted in a low amount of fecal material. Therefore, DNA extraction for genomic analysis was carried out on 35 samples. Hybridization was performed for 22 h and cartridges were posed into the prep station for 3 h washing and then read to the digital counter for a further 5 h. Then *n*-solver software was used for the analysis

Study design and analytical workflow are shown in Fig. 1. Subjects enrolled were divided into two groups: healthy-negative for fecal occult blood (herein indicated as N-FOB; *n* = 48) and positive for fecal occult blood (herein indicated as P-FOB; *n* = 48). DNA extraction and/or genomic analysis was carried out for N-FOB *n* = 35 and P-FOB *n* = 35 samples, due to low amount of starting material.

Clinical data are reported in Table 1. The clinical trial was registered at https://clinicaltrials.gov/ct2/show/NCT03388424. This study was authorized by the ethical committee of University of Catanzaro (protocol #287, November 2017) and informed consent was obtained from each patient. All procedures were conducted according to the principles expressed in the Declaration of Helsinki and the Guideline for Good Clinical Practice.

**Table 1:** Clinical data of enrolled subjects

Sample	Age (years)	Male	Female	Blood amount in stool sample and its percentage
N-FOB	Range: 30–70	18	30	Absence of occult blood in stool
P-FOB*	Range: 50–70	23	25	− 100 to 300 ng/ml: 82% − 300 to 600 ng/ml: 10% > 800 ng/ml: 8%

*Data from f-Hb (OC-Sensor Diana-Eiken Chemical-Tokyo)

0.5–1 g of fresh feces were collected in OMNI gene-GUT OMR 200 (DNA Genotek Inc, Ottawa, Canada). The stool samples were then carefully mixed with 2 mL of stabilization buffer in the provided tube and stored at room temperature.

### DNA extraction and quantification

DNA extraction was performed by automatic system MagCore HF16 Plus. Briefly, 2 mL of feces samples was first exposed to 2 h of lysozyme (final concentration 250 µg/ml) treatment at 37 °C, then digested with Proteinase K solution (10 mg/ml, in GT buffer) at 65 °C for 3 h. Samples were centrifuged and the bacterial pellet was used for microbial DNA isolation by MagCore protocol cartridge 401 and carrier-RNA or cartridge 202.

The DNA quantity and quality measurements were performed on Qubit 3.0 using the Qubit dsDNA HS (High Sensitivity Assay by Thermo Fisher Scientific), based on the fluorescence readouts.

### Microbiome panel design, NanoString sample preparation and nSolver™ rcc file acquisition

Genomic DNA ID from Taxonomy for 16S rRNA gene of 79 bacterial strains was identified, of which three are not present in the human gut and three belong to the host DNA. The panel details are reported in Supplementary Table 1.

For the n-counter flex DX of NanoString Technology, 400 ng of 16S rRNA gene was used as input. After 2 h of AluI digestion at 37 °C, the sample was then hybridized with CodeSet (Supplementary File 1) for 22 h at 65 °C.

The unhybridized CodeSet was removed with automated purification performed on an nCounter Prep Station, and the remaining target probe complexes were transferred and bound to an imaging surface as previously described (Panek et al. 2018; Geiss et al. 2008). Counts of the two reporter probes were tabulated for each sample by the nCounter Digital Analyzer.

### NanoString reproducibility, robustness and Clostridium difficile testing with GeneExpert–Cepheid™

The reproducibility of our set of experimental tests was monitored through the negative (AH # 8) and positive (AF # 6) control probes include in the panel by NanoString, as well as probe value for 12 samples were obtained with two different n-counter flex machine included in the panel and reported in the Supplementary Table 3 as count numbers. The robustness of the technology for the clinical sample was already studied (Veldman-Jones et al. 2015). Finally, to corroborate the proposed experiments, some samples were randomly (1:4) analyzed with a common technology (GeneExpert—Cepheid GXCDIFFBT-CE-10) for *Clostridium difficile* used in molecular microbiology clinical practice (Supplementary Fig. 1).

### Statistical analysis

Unless indicated, statistical significance was determined by a two-tailed Student’s *t* test with suitable multiple comparison correction. *p* value of < 0.05 was regarded as significant. Results are expressed as mean ± SD. A coefficient of variation (CV) of 60% was chosen as cutoff into the *n*-solver software analysis. CV is used for comparison between data sets with different units or widely different means. One sample from healthy subjects (N-FOB) was excluded due to the presence of a warning red flag in the n-solver analysis.

## Results and discussion

The complex interaction between organism and microbiome, in both physiological and pathophysiological conditions, has attracted interest from the scientific community either for personalized medicine or to develop probiotics supplement to relieve gut nuisance (Salazar et al. 2017; Wu and Wu 2012; Mu et al. 2016; Wen and Duffy 2017; Seo et al. 2013; Dahiya et al. 2017; Yadav et al. 2018). Furthermore, intestinal discomfort is characterized by pain and gut inflammation. The panel design was fulfilled for intestinal discomfort and according to the scientific literature (Chichlowski and Rudolph 2015; Simrén et al. 2013).

Currently in the experimental pipeline of 16S rRNA gene sequence with HTS, each procedural step introduces a variation that could influence the final output (Pollock et al. 2018). Therefore, there is an unmet need for standardization of methodology which would enable a reliable and reproducible analysis of valuable human biological samples for studying gut microbiota (Pollock et al. 2018; Rintala et al. 2017; Panek et al. 2018).

**Fig. 2:**
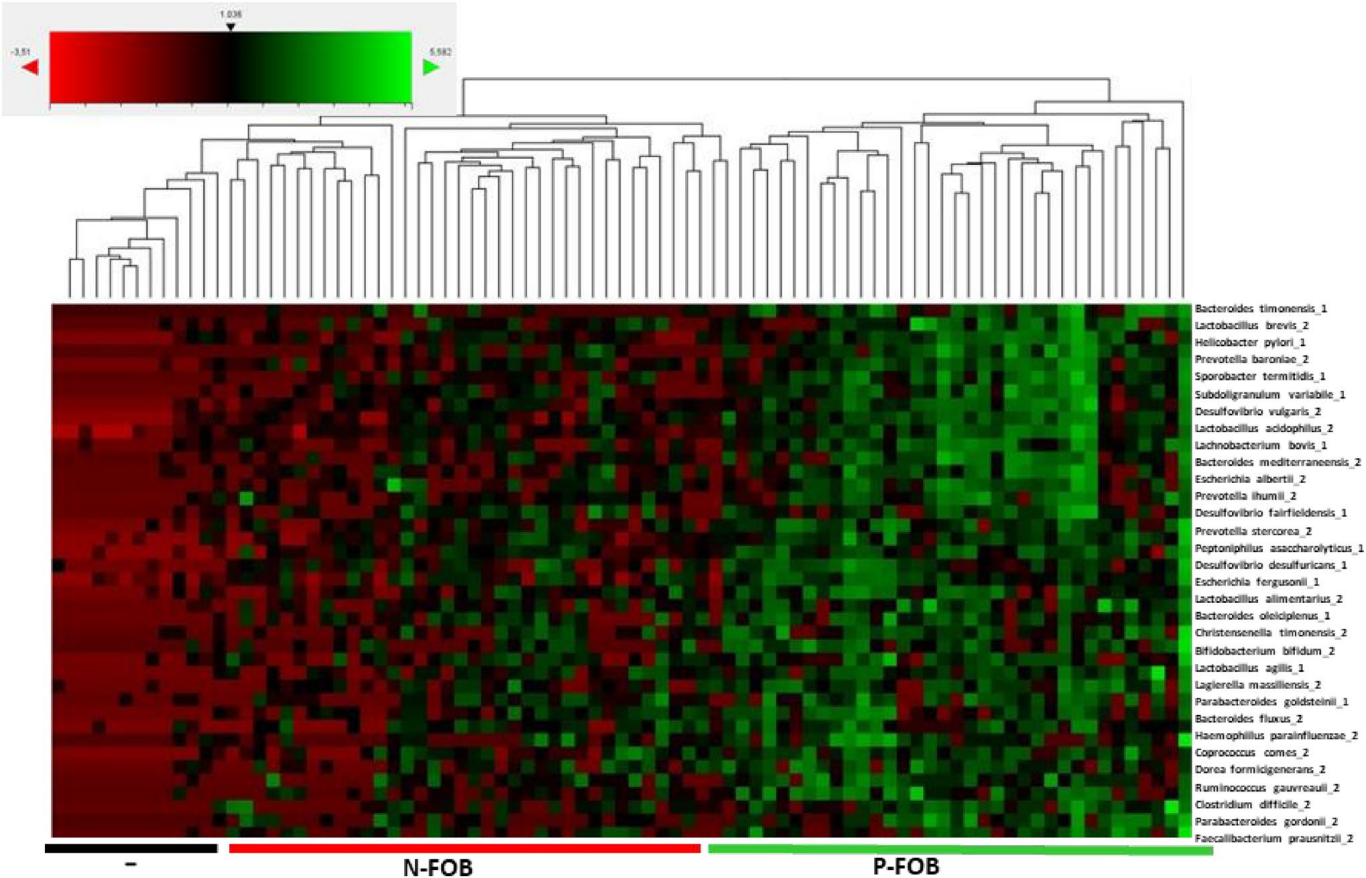
Heat map and hierarchical clustering of N-FOB (*n* = 34), and P-FOB (*n* = 35) based on the differentially present bacteria 16S rRNA in log2 ratios. The color intensity represents changes in bacteria variation, ranging from − 3.51 to 5.582. In the analysis, red represents low bacteria abundance and green represents high bacteria abundance. Black represents unchanged bacteria presence as evident by the color reference. *n*-Solver software was used. A coefficient of variation of 60% was applied

Herein, we show that the direct detection of 16S rRNA target gene via hybridization method allows us to appreciate the variation of biodiversity (Fig. 2 and Supplementary Table 2) within the collected samples (Table 1). The designed panel based on 16S rRNA gene was suitable for n-counter flex platform considering also the haploid nature of the bacteria DNA (Geiss et al. 2008; Griswold 2018).

The minimum input of nucleic acid used in this study was 400 ng. The DNA extraction was performed as described in “Materials and methods”. The highest DNA yields (an enrichment of DNA from 40- to 48-fold) were obtained using the cartridge 401 modified protocol compared to cartridge 202 (Table 2).

**Table 2:** Comparison of total DNA extraction from stool samples between 202 (G1) and 401 (G2) cartridge protocols

G1	Total DNA ng/µl # 202 cartridge	G2	Total DNA ng/µl # 401 cartridge	Enrichment G2 vs G1	*p* value
1	1.5227 ± 0.0148	1	61.8962 ± 0.0176***	40.65	< 0.001
2	1.3951 ± 0.0080	2	62.4069 ± 0.0142***	44.73	< 0.001
3	1.2539 ± 0.0093	3	60.5769 ± 0.0167***	48.31	< 0.001
4	1.0769 ± 0.0092	4	52.1298 ± 0.0212***	48.40	< 0.001

16S rRNA gene array profiles generate a heat map and hierarchical clustering based on the most differentiated biodiversity as shown in Fig. 2. The analyzed data set is composed of 5.530 count number of 16S rRNA target, related to 79 bacteria recognized by the best hybridization probe and detected in 69 different subjects.

The samples were classified according to two clusters: P-FOB (in green) and N-FOB (in red) negative control (in black). Of 79 bacteria, 32 displayed variation between the two clusters. The differential bacteria distribution in P-FOB compared to N-FOB showed an increased presence of part of them in P-FOB subjects. The agglomerative cluster of the heat map with a dendrogram tree showed an obvious clustering of 16S rRNA-specific bacteria genes that ranged from − 3.51 up to 5.582 in P-FOB subjects, as illustrated in Fig. 2. Red indicates decrease up to − 3.51 of 16S rRNA-specific bacteria genes, while green indicates an increase up to 5.582 of 16S rRNA-specific bacteria genes.

Data from the heat map, reported in Fig. 2 and in Supplementary Table 2, show that bacteria biodiversity between the two groups is greatly different. The information obtained is in agreement with those reported in a recently paper from Tarallo et al. (2019).

In particular, bacteria belonging to Bacteroidetes phylum was recently reviewed for its role in metabolic disease, among which *Prevotella* assumes an important meaning (Johnson et al. 2017). Likewise, Proteobacteria phylum such as *Helicobacter pylori*, *Desulfovibrio vulgaris*, *Desulfovibrio fairfieldensis,* *Desulfovibrio desulfuricans*, *Escherichia fergusonii*, and *Haemophiilus parainfluenzae* are higher in pathophysiological gut status according to our heat map results and previous studies (Tarallo et al. 2019; Rizzatti et al. 2017). Additionally, Libby et al. (2019) pointed out a significant correlation for fecal blood presence and the increasing risk of dying from circulatory, respiratory and digestive diseases (excluding colorectal cancer) as well as neuropsychological, blood and endocrine disease (Walker 1990). Therefore, defining the nature of dysbiosis for the presence of blood in feces could prevent these risks also combining biotechnological intervention (Khangwal and Shukla 2019). The observations described here have two possible implications concerning gut dysbiosis. First, the dysbiosis in the microbiome linked to blood feces presence might also be used to explore the underlying reasons for different patterns of mortality in different populations across the world. Second, the proper prebiotics/probiotics intervention could modify microbiome dysbiosis and possibly blood feces biomarker to reduce the risk of premature mortality.

In addition, *Firmicutes* phylum abundances were significantly different in cancer stool sample compared to healthy or the adenoma sample (Tarallo et al. 2019). In this context,, results from our trial showed a higher presence of *Clostridium difficile* that displayed about 70% of low presence in N-FOB compared to P-FOB subjects (21/34 red dots of N-FOB) vs 24/35 green dots in P-FOB). These data assume particular interest in preventing *Clostridium difficile* infection, where in extreme condition fecal microbiome transplantation can occur (Juul et al. 2018). The importance of testing microbiome is becoming more evident, especially considering that the gut axis interaction involves several organs (brain, kidney, liver, bone, skin, adipose tissue and heart) (Ahlawat and Sharma 2020). Consequently, comprehensive information of the types of microbes that reside in the human gut is necessary before any kind of pharmacological intervention that attempts to alter the microbiome. Our methods could be applied successfully on long-term archived fecal sample sets, originally collected for testing fecal blood, to stratify patients and could be used for microbiome-based early biomarker discovery for gut health (Rounge et al. 2018). Of note, this is the first time that direct hybridization with n-counter flex DX platform was applied to microbiome studies and although second generation of platform already exists, both produced equivalent signals and signal deviations (Yu et al. 2019). Finally, it is important to underline that using two different n-counter flex machines, we obtained similar results (*p* > 0.05) showing both, reproducibility and robustness of the n-counter technology. Moreover, comparing the results obtained using a microbiological clinical diagnostic tool for* Clostridium difficile* with the results obtained from n-counter flex DX, the presence of this bacterium in P-FOB subjects was successfully reported, corroborating our results.

## Conclusions

The complex interaction between organism and microbiome, both in the physiology and in the pathophysiology, has aroused much interest in the last years. The microbiome represents one of the most significant new topics in the biomedical field that has concretely entered the medical/therapeutic field. This is the first study undertaken to determine HMB by direct hybridization using n-counter flex DX technology. This approach gives a useful tool for robust diagnostic/screening profiles of the microbiome. It is an innovative and exportable diagnostic model in the laboratory medicine practice. Furthermore, as the HMB panel could have strength up to 800 bacteria, this technology could lead to new biomarkers’ discovery of microbiome and pave the way for the identification of therapeutic targets for human well-being.

## Electronic supplementary material

Below is the link to the electronic supplementary material.

Supplementary file1 (XLSX 29 kb)

Supplementary file2 (DOCX 235 kb)

Supplementary file3 (XLSX 853 kb)

Supplementary file4 (DOCX 17 kb)

Supplementary file5 (DOCX 29 kb)

## Data Availability

Material and raw data are available.

## References

[CR1] Ahlawat S, Sharma KK (2020). Gut–organ axis: a microbial outreach and networking. Lett Appl Microbiol.

[CR2] Chichlowski M, Rudolph C (2015). Visceral pain and gastrointestinal microbiome. J Neurogastroenterol Motil.

[CR3] Clooney AG, Fouhy F, Sleator RD, O'Driscoll A, Stanton C, Cotter PD, Claesson MJ (2016). Comparing apples and oranges?: next generation sequencing and its impact on microbiome analysis. PLoS One.

[CR4] Dahiya DK, Renuka PM, Shandilya UK, Dhewa T, Kumar N, Kumar S, Puniya AK, Shukla P (2017). Gut microbiota modulation and its relationship with obesity using prebiotic fibers and probiotics: a review. Front Microbiol.

[CR5] D'Amore R, Ijaz UZ, Schirmer M, Kenny JG, Gregory R, Darby AC, Shakya M, Podar M, Quince C, Hall N (2016). A comprehensive benchmarking study of protocols and sequencing platforms for 16s rRNA community profiling. BMC Genomics.

[CR6] Fischbach MA, Segre JA (2016). Signaling in host-associated microbial communities. Cell.

[CR7] Geiss GK, Bumgarner RE, Birditt B, Dahl T, Dowidar N, Dunaway DL, Fell HP, Ferree S, George RD, Grogan T (2008). Direct multiplexed measurement of gene expression with color-coded probe pairs. Nat Biotechnol.

[CR8] Griswold A (2018). Genome packaging in prokaryotes: the circular chromosome of *E. Coli*. Nat Educ.

[CR9] Johnson EL, Heaver SL, Walters WA, Ley RE (2017). Microbiome and metabolic disease: revisiting the bacterial phylum Bacteroidetes. J Mol Med (Berl).

[CR10] Juul FE, Garborg K, Bretthauer M, Skudal H, Øines MN, Wiig H, Rose Ø, Seip B, Lamont JT, Midtvedt T, Valeur J, Kalager M (2018). Fecal microbiota transplantation for primary *Clostridium difficile* infection. N Engl J Med.

[CR11] Karl JP, Hatch AM, Arcidiacono SM, Pearce SC, Pantoja-Feliciano IG, Doherty LA, Soares JW (2018). Effects of psychological, environmental and physical stressors on the gut microbiota. Front Microbiol.

[CR12] Kastl AJ, Terry NA, Wu GD, Albenberg LG (2020). The structure and function of the human small intestinal microbiota: current understanding and future directions. Cell Mol Gastroenterol Hepatol.

[CR13] Khangwal I, Shukla P (2019). Combinatory biotechnological intervention for gut microbiota. Appl Microbiol Biotechnol.

[CR14] Lam HY, Clark MJ, Chen R, Chen R, Natsoulis G, O'Huallachain M, Dewey FE, Habegger L, Ashley EA, Gerstein MB, Butte AJ, Ji HP, Snyder M (2011). Performance comparison of whole-genome sequencing platforms. Nat Biotechnol.

[CR15] Li X, Wu S, Du Y, Yang L, Li Y, Hong B (2020). Entecavir therapy reverses gut microbiota dysbiosis induced by hepatitis B virus infection in a mouse model. Int J Antimicrob Agents.

[CR16] Libby G, Fraser CG, Carey FA, Brewster DH, Steele RJC (2018). Occult blood in faeces is associated with all-cause and non-colorectal cancer mortality. Gut.

[CR17] Loman NJ, Misra RV, Dallman TJ, Constantinidou C, Gharbia SE, Wain J, Pallen MJ (2012). Performance comparison of benchtop high-throughput sequencing platforms. Nat Biotechnol.

[CR18] Marchesi JR, Adams DH, Fava F, Hermes GD, Hirschfield GM, Hold G, Quraishi MN, Kinross J, Smidt H, Tuohy KM, Thomas LV, Zoetendal EG, Hart A (2016). The gut microbiota and host health: a new clinical frontier. Gut.

[CR19] Mohammadi A, Kelly OB, Smith MI, Kabakchiev B, Silverberg MS (2019). Differential miRNA expression in ileal and colonic tissues reveals an altered immunoregulatory molecular profile in individuals with Crohn's disease versus healthy subjects. J Crohns Colitis.

[CR20] Mu C, Yang Y, Zhu W (2016). Gut microbiota: the brain peacekeeper. Front Microbiol.

[CR21] Nagpal R, Mainali R, Ahmadi S, Wang S, Singh R, Kavanagh K, Kitzman DW, Kushugulova A, Marotta F, Yadav H (2018). Gut microbiome and aging: physiological and mechanistic insights. Nutr Healthy Aging.

[CR22] Panek M, Čipčić Paljetak H, Barešić A, Perić M, Matijašić M, Lojkić I, Vranešić Bender D, Krznarić Ž, Verbanac D (2018). Methodology challenges in studying human gut microbiota - effects of collection, storage, DNA extraction and next generation sequencing technologies. Sci Rep.

[CR23] Pollock J, Glendinning L, Wisedchanwet T, Watson M (2018). The madness of microbiome: attempting to find consensus "best practice" for 16S microbiome studies. Appl Environ Microbiol.

[CR24] Quail MA, Smith M, Coupland P, Otto TD, Harris SR, Connor TR, Bertoni A, Swerdlow HP, Gu Y (2012). A tale of three next generation sequencing platforms: comparison of Ion Torrent, Pacific Biosciences and Illumina MiSeq sequencers. BMC Genom.

[CR25] Rajilić-Stojanović M, de Vos WM (2014). The first 1000 cultured species of the human gastrointestinal microbiota. FEMS Microbiol Rev.

[CR26] Rintala A, Pietilä S, Munukka E, Eerola E, Pursiheimo JP, Laiho A, Pekkala S, Huovinen P (2017). Gut microbiota analysis results are highly dependent on the 16S rRNA gene target region, whereas the impact of DNA extraction is minor. J Biomol Tech.

[CR27] Rizzatti G, Lopetuso LR, Gibiino G, Binda C, Gasbarrini A (2017). Proteobacteria: a common factor in human diseases. Biomed Res Int.

[CR28] Rounge TB, Meisal R, Nordby JI, Ambur OH, de Lange T, Hoff G (2018). Evaluating gut microbiota profiles from archived fecal samples. BMC Gastroenterol.

[CR29] Salazar N, Valdés-Varela L, González S, Gueimonde M, de Los Reyes-Gavilán CG (2017). Nutrition and the gut microbiome in the elderly. Gut Microbes.

[CR30] Salipante SJ, Kawashima T, Rosenthal C, Hoogestraat DR, Cummings LA, Sengupta DJ, Harkins TT, Cookson BT, Hoffman NG (2016). Erratum for Salipante et al., Performance comparison of illumina and ion torrent next-generation sequencing platforms for 16S rRNA-based bacterial community profiling. Appl Environ Microbiol..

[CR31] Seo AY, Kim N, Oh DH (2013). Abdominal bloating: pathophysiology and treatment. J Neurogastroenterol Motil.

[CR32] Simrén M, Barbara G, Flint HJ, Spiegel BM, Spiller RC, Vanner S, Verdu EF, Whorwell PJ, Zoetendal EG, Committee RF (2013). Intestinal microbiota in functional bowel disorders: a Rome foundation report. Gut.

[CR33] Tarallo S, Ferrero G, Gallo G, Francavilla A, Clerico G, Realis Luc A, Manghi P, Thomas AM, Vineis P, Segata N, Pardini B, Naccarati A, Cordero F (2019). Altered fecal small RNA profiles in colorectal cancer reflect gut microbiome composition in stool samples. mSystems..

[CR34] Thursby E, Juge N (2017). Introduction to the human gut microbiota. Biochem J.

[CR35] Veldman-Jones MH, Brant R, Rooney C (2015). Evaluating robustness and sensitivity of the nanostring technologies nCounter platform to enable multiplexed gene expression analysis of clinical samples. Cancer Res.

[CR36] Walker HK, Walker HK, Hall WD, Hurst JW (1990). The origins of the history and physical examination. Clinical methods: the history, physical, and laboratory examinations.

[CR37] Wen L, Duffy A (2017). Factors influencing the gut microbiota, inflammation, and type 2 diabetes. J Nutr.

[CR38] Wu HJ, Wu E (2012). The role of gut microbiota in immune homeostasis and autoimmunity. Gut Microbes.

[CR39] Yadav R, Kumar V, Baweja M, Shukla P (2018). Gene editing and genetic engineering approaches for advanced probiotics: a review. Crit Rev Food Sci Nutr.

[CR40] Yu L, Bhayana S, Jacob NK, Fadda P (2019). Comparative studies of two generations of NanoString nCounter system. PLoS One.

