# Supplementary material for: Dysbiosis in intestinal microbiome linked to fecal blood determined by direct hybridization
Source: 3 Biotech. 2020 Jul 28;10(8):358. doi: 10.1007/s13205-020-02351-w (PMC7387388; doi:10.1007/s13205-020-02351-w)
Supplement: Supplementary file 2 — Supplementary file2 (DOCX 235 kb) [file 13205_2020_2351_MOESM2_ESM.docx]

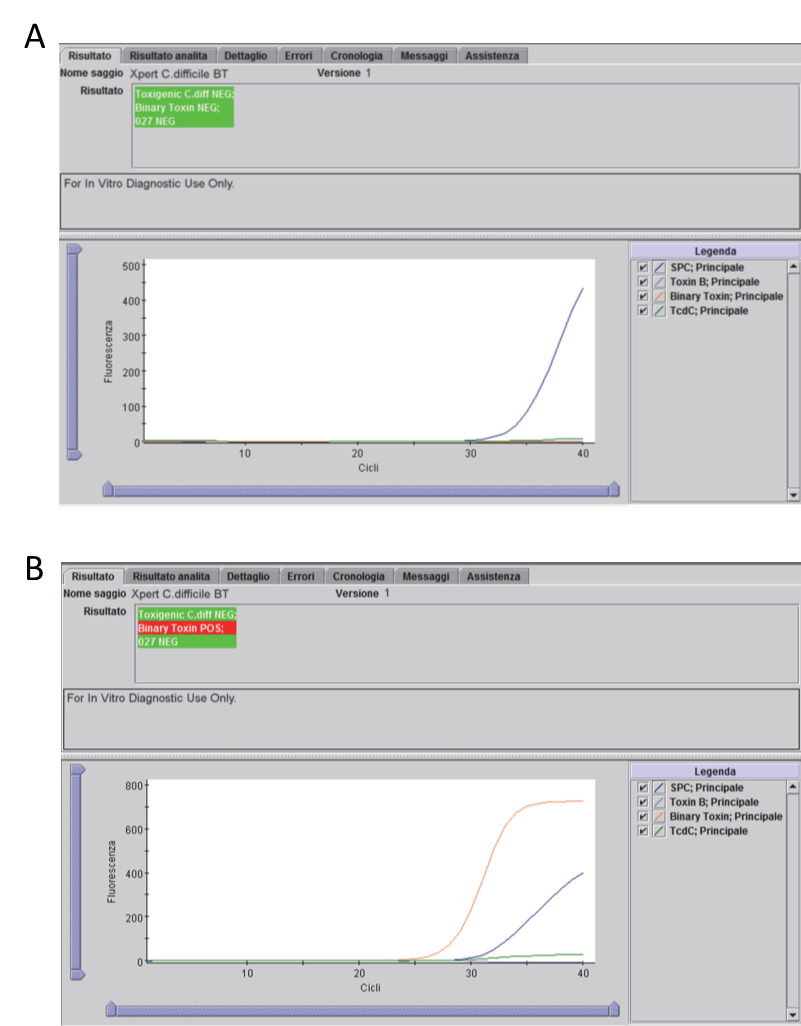


**Supplementary Figure 1**: Representative image of A) N-FOB and B) P-FOB samples analyzed with GeneExpert –Cepheid GXCDIFFBT-CE-10 for *Clostridium difficile*. Each test includes a Sample Processing Control (SPC).
