# Supplementary material for: Dysbiosis in intestinal microbiome linked to fecal blood determined by direct hybridization
Source: 3 Biotech. 2020 Jul 28;10(8):358. doi: 10.1007/s13205-020-02351-w (PMC7387388; doi:10.1007/s13205-020-02351-w)
Supplement: Supplementary file 4 — Supplementary file4 (DOCX 17 kb) [file 13205_2020_2351_MOESM4_ESM.docx]

| **Bacteria name** | **NCBI TAX ID** | **ncbi link** |
| --- | --- | --- |
|  |  |  |
| Abiotrophia defectiva | 46125 | <https://www.ncbi.nlm.nih.gov/genome/?term=Abiotrophia+defectiva> |
| Akkermansia glycaniphila | 1679444 | <https://www.ncbi.nlm.nih.gov/genome/?term=Akkermansia+glycaniphila> |
| Akkermansia muciniphila | 239935 | <https://www.ncbi.nlm.nih.gov/genome/?term=Akkermansia+muciniphila> |
| Alistipes putredinis | 28117 | <https://www.ncbi.nlm.nih.gov/genome/?term=Alistipes+putredinis> |
| Anaerotruncus colihominis | 169435 | <https://www.ncbi.nlm.nih.gov/genome/?term=Anaerotruncus+colihominis> |
| Bacteroides caccae | 47678 | <https://www.ncbi.nlm.nih.gov/genome/?term=Bacteroides+caccae> |
| Bacteroides cellulosilyticus | 246787 | <https://www.ncbi.nlm.nih.gov/genome/?term=Bacteroides+cellulosilyticus> |
| Bacteroides clarus | 626929 | <https://www.ncbi.nlm.nih.gov/genome/?term=Bacteroides+clarus> |
| Bacteroides coprocola | 310298 | <https://www.ncbi.nlm.nih.gov/genome/?term=Bacteroides+coprocola> |
| Bacteroides coprophilus | 387090 | <https://www.ncbi.nlm.nih.gov/genome/?term=Bacteroides+coprophilus> |
| Bacteroides dorei | 357276 | <https://www.ncbi.nlm.nih.gov/genome/?term=Bacteroides+dorei> |
| Bacteroides eggerthii | 28111 | <https://www.ncbi.nlm.nih.gov/genome/?term=Bacteroides+eggerthii> |
| Bacteroides faecis | 674529 | <https://www.ncbi.nlm.nih.gov/genome/?term=Bacteroides+faecis> |
| Bacteroides fluxus | 626930 | <https://www.ncbi.nlm.nih.gov/genome/?term=Bacteroides+fluxus> |
| Bacteroides fragilis | 817 | <https://www.ncbi.nlm.nih.gov/genome/?term=Bacteroides+fragilis> |
| Bacteroides intestinalis | 329854 | <https://www.ncbi.nlm.nih.gov/genome/?term=Bacteroides+intestinalis> |
| Bacteroides mediterraneensis | 1841856 | <https://www.ncbi.nlm.nih.gov/genome/?term=Bacteroides+mediterraneensis> |
| Bacteroides oleiciplenus | 626931 | <https://www.ncbi.nlm.nih.gov/genome/?term=Bacteroides%20oleiciplenus> |
| Bacteroides ovatus | 28116 | <https://www.ncbi.nlm.nih.gov/genome/?term=Bacteroides+ovatus> |
| Bacteroides plebeius | 310297 | <https://www.ncbi.nlm.nih.gov/genome/?term=Bacteroides+plebeius> |
| Bacteroides stercoris | 46506 | https://www.ncbi.nlm.nih.gov/genome/?term=Bacteroides+stercoris |
| Bacteroides timonensis | 1470345 | https://www.ncbi.nlm.nih.gov/genome/?term=Bacteroides+timonensis |
| Bacteroides vulgatus | 821 | https://www.ncbi.nlm.nih.gov/genome/?term=Bacteroides+vulgatus |
| Bifidobacterium adolescentis | 1680 | https://www.ncbi.nlm.nih.gov/genome/?term=Bifidobacterium+adolescentis |
| Bifidobacterium bifidum | 1681 | https://www.ncbi.nlm.nih.gov/genome/?term=Bifidobacterium+bifidum |
| Bifidobacterium breve | 1685 | https://www.ncbi.nlm.nih.gov/genome/?term=Bifidobacterium+breve |
| Bifidobacterium longum | 216816 | https://www.ncbi.nlm.nih.gov/genome/?term=Bifidobacterium+longum |
| Blautia hydrogenotrophica | 53443 | https://www.ncbi.nlm.nih.gov/genome/?term=Blautia+hydrogenotrophica |
| Christensenella massiliensis | 1805714 | https://www.ncbi.nlm.nih.gov/genome/?term=Christensenella+massiliensis |
| Christensenella minuta | 626937 | https://www.ncbi.nlm.nih.gov/genome/?term=Christensenella+minuta |
| Christensenella timonensis | 1816678 | https://www.ncbi.nlm.nih.gov/genome/?term=Christensenella+timonensis |
| Clostridium difficile | 1496 | https://www.ncbi.nlm.nih.gov/genome/?term=Clostridium+difficile |
| Collinsella aerofaciens | 74426 | https://www.ncbi.nlm.nih.gov/genome/?term=Collinsella+aerofaciens |
| Coprococcus catus | 116085 | <https://www.ncbi.nlm.nih.gov/genome/?term=Coprococcus+catus> |
| Coprococcus comes | 410072 | https://www.ncbi.nlm.nih.gov/genome/?term=Coprococcus+comes |
| Coprococcus eutactus | 33043 | https://www.ncbi.nlm.nih.gov/genome/?term=Coprococcus+eutactus |
| Desulfovibrio desulfuricans | 876 | https://www.ncbi.nlm.nih.gov/genome/?term=Desulfovibrio+desulfuricans |
| Desulfovibrio fairfieldensis | 44742 | https://www.ncbi.nlm.nih.gov/genome/?term=Desulfovibrio+fairfieldensis |
| Desulfovibrio piger | 901 | https://www.ncbi.nlm.nih.gov/genome/?term=Desulfovibrio+piger |
| Desulfovibrio vulgaris | 881 | https://www.ncbi.nlm.nih.gov/genome/?term=Desulfovibrio+vulgaris |
| Dorea formicigenerans | 39486 | https://www.ncbi.nlm.nih.gov/genome/?term=dorea+formicigenerans%5Borgn%5D |
| Escherichia albertii | 208962 | https://www.ncbi.nlm.nih.gov/genome/?term=Escherichia+albertii |
| Escherichia coli | 562 | https://www.ncbi.nlm.nih.gov/genome/?term=Escherichia+coli |
| Escherichia fergusonii | 564 | https://www.ncbi.nlm.nih.gov/genome/?term=Escherichia+fergusonii |
| Faecalibacterium prausnitzii | 853 | https://www.ncbi.nlm.nih.gov/genome/?term=Faecalibacterium+prausnitzii |
| Finegoldia magna ATCC 29328 | 334413 | https://www.ncbi.nlm.nih.gov/genome/?term=Finegoldia+magna+ATCC+29328 |
| Haemophiilus parainfluenzae | 729 | https://www.ncbi.nlm.nih.gov/genome/?term=Haemophiilus+parainfluenzae |
| Helicobacter pylori | 210 | https://www.ncbi.nlm.nih.gov/genome/?term=Helicobacter+pylori |
| Lachnobacterium bovis | 140626 | https://www.ncbi.nlm.nih.gov/genome/?term=Lachnobacterium+bovis |
| Lactobacillus acidophilus | 1579 | https://www.ncbi.nlm.nih.gov/genome/?term=Lactobacillus+acidophilus |
| Lactobacillus agilis | 1601 | https://www.ncbi.nlm.nih.gov/genome/?term=Lactobacillus+agilis |
| Lactobacillus alimentarius | 1602 | https://www.ncbi.nlm.nih.gov/genome/?term=Lactobacillus+alimentarius |
| Lactobacillus brevis | 1580 | https://www.ncbi.nlm.nih.gov/genome/?term=Lactobacillus+brevis |
| Lactobacillus crispatus | 477770 | https://www.ncbi.nlm.nih.gov/genome/?term=Lactobacillus+crispatus |
| Lactobacillus gasseri | 1596 | https://www.ncbi.nlm.nih.gov/genome/?term=Lactobacillus+gasseri |
| Lactobacillus rhamnosus | 47715 | https://www.ncbi.nlm.nih.gov/genome/?term=Lactobacillus+rhamnosus |
| Lactobacillus salivarius | 1624 | https://www.ncbi.nlm.nih.gov/genome/1207 |
| Lagierella massiliensis | 1689303 | <https://www.ncbi.nlm.nih.gov/genome/?term=Lagierella+massiliensis> |
| Parabacteroides distasonis | 823 | https://www.ncbi.nlm.nih.gov/genome/?term=Parabacteroides+distasonis |
| Parabacteroides faecis | 1217282 | https://www.ncbi.nlm.nih.gov/pubmed/25667391 |
| Parabacteroides goldsteinii | 328812 | https://www.ncbi.nlm.nih.gov/genome/?term=Parabacteroides+goldsteinii |
| Parabacteroides gordonii | 574930 | <https://www.ncbi.nlm.nih.gov/genome/?term=Parabacteroides+gordonii> |
| Parabacteroides johnsonii | 387661 | <https://www.ncbi.nlm.nih.gov/genome/?term=Parabacteroides+johnsonii> |
| Parabacteroides merdae | 46503 | <https://www.ncbi.nlm.nih.gov/genome/?term=Parabacteroides+merdae> |
| Peptoniphilus asaccharolyticus | 1258 | https://www.ncbi.nlm.nih.gov/genome/?term=Peptoniphilus+asaccharolyticus |
| Prevotella baroniae | 305719 | https://www.ncbi.nlm.nih.gov/genome/?term=Prevotella+baroniae |
| Prevotella copri | 165179 | https://www.ncbi.nlm.nih.gov/genome/?term=Prevotella+copri |
| Prevotella ihumii | 1917878 | https://www.ncbi.nlm.nih.gov/genome/?term=Prevotella+ihumii |
| Prevotella stercorea | 363265 | https://www.ncbi.nlm.nih.gov/genome/?term=Prevotella+stercorea |
| Roseburia intestinalis | 166486 | https://www.ncbi.nlm.nih.gov/genome/?term=Roseburia+intestinalis |
| Ruminococcus faecis | 592978 | https://www.ncbi.nlm.nih.gov/genome/?term=Ruminococcus+faecis |
| Ruminococcus gauvreauii | 438033 | https://www.ncbi.nlm.nih.gov/genome/?term=Ruminococcus+gauvreauii |
| Ruminococcus gnavus | 33038 | https://www.ncbi.nlm.nih.gov/genome/?term=Ruminococcus+gnavus |
| Ruminococcus torques | 33039 | https://www.ncbi.nlm.nih.gov/genome/?term=Ruminococcus+torques |
| Sporobacter termitidis | 44749 | https://www.ncbi.nlm.nih.gov/genome/?term=Sporobacter+termitidis |
| Streptococcus anginosus | 1328 | https://www.ncbi.nlm.nih.gov/genome/?term=Streptococcus+anginosus |
| Subdoligranulum variabile | 214851 | https://www.ncbi.nlm.nih.gov/genome/?term=Subdoligranulum+variabile |
| ACTB | HUMAN GENE |  |
| GAPDH | HUMAN GENE |  |
| HDAC3 | HUMAN GENE |  |
| Alicyclobacillus acidiphilus | Not in human gut | https://www.ncbi.nlm.nih.gov/genome/?term=Alicyclobacillus+acidiphilus |
| Rhizobium radiobacter | Not in human gut | https://www.ncbi.nlm.nih.gov/genome/?term=Rhizobium+radiobacter |
| Salinibacter ruber | Not in human gut | https://www.ncbi.nlm.nih.gov/genome/?term=Salinibacter+ruber |

**Supplementary Table 1.** Name of bacteria, NCBI TAX ID and relative link.
