# Supplementary material for: Dysbiosis in intestinal microbiome linked to fecal blood determined by direct hybridization
Source: 3 Biotech. 2020 Jul 28;10(8):358. doi: 10.1007/s13205-020-02351-w (PMC7387388; doi:10.1007/s13205-020-02351-w)
Supplement: Supplementary file 5 — Supplementary file5 (DOCX 29 kb) [file 13205_2020_2351_MOESM5_ESM.docx]

| **HeatMap Data** | | | | | | | | | | | | | | | | |
| --- | --- | --- | --- | --- | --- | --- | --- | --- | --- | --- | --- | --- | --- | --- | --- | --- |
| **ID-PROBE** |  | | | | | | | **ID-PROBE** |  | | | | | | | |
| BT1 | 1° line | **N-FOB** |  | #dots/tot | **P-FOB** |  | #dots/tot | LB2 | 2°line | **N-FOB** |  | #dots/tot |  | **P-FOB** |  | #dots/tot |
|  |  | green |  | 3/34 | green |  | 21/35 |  |  | green |  | 10/34 |  | green |  | 16/35 |
|  |  | red |  | 26/34 | red |  | 8/35 |  |  | red |  | 13/34 |  | red |  | 6/35 |
|  |  | black |  | 5/34 | black |  | 6/35 |  |  | black |  | 11/34 |  | black |  | 13/35 |
| HP1 | 3°line |  |  |  |  |  |  | PB2 | 4°line |  |  |  |  |  |  |  |
|  |  | green |  | 5/34 | green |  | 25/35 |  |  | green |  | 9/34 |  | green |  | 31/35 |
|  |  | red |  | 13/34 | red |  | 3/35 |  |  | red |  | 20/34 |  | red |  | 3/35 |
|  |  | black |  | 16/34 | black |  | 7/35 |  |  | black |  | 5/34 |  | black |  | 1/35 |
| ST1 | 5°line |  |  |  |  |  |  | SV1 | 6°line |  |  |  |  |  |  |  |
|  |  | green |  | 11/34 | green |  | 28/35 |  |  | green |  | 6/34 |  | green |  | 24/35 |
|  |  |  |  |  |  |  |  |  |  |  |  |  |  |  |  |  |
|  |  | red |  | 20/34 | red |  | 4/35 |  |  | red |  | 25/34 |  | red |  | 4/35 |
|  |  | black |  | 3/34 | black |  | 3/35 |  |  | black |  | 3/34 |  | black |  | 7/35 |
| DV2 | 7°line |  |  |  |  |  |  | LA2 | 8°line |  |  |  |  |  |  |  |
|  |  | green |  | 10/34 | green |  | 28/35 |  |  | green |  | 8/34 |  | green |  | 25/35 |
|  |  | red |  | 18/34 | red |  | 5/35 |  |  | red |  | 17/34 |  | red |  | 6/35 |
|  |  | black |  | 6/34 | black |  | 2/35 |  |  | black |  | 9/34 |  | black |  | 4/35 |
| LB1 | 9°line |  |  |  |  |  |  | BM2 | 10°line |  |  |  |  |  |  |  |
|  |  | green |  | 10/34 | green |  | 30/35 |  |  | green |  | 9/34 |  | green |  | 29/35 |
|  |  | red |  | 18/34 | red |  | 2/35 |  |  | red |  | 22/34 |  | red |  | 5/35 |
|  |  | black |  | 6/34 | black |  | 3/35 |  |  | black |  | 3/34 |  | black |  | 1/35 |
| EA2 | 11°line |  |  |  |  |  |  | PI2 | 12°line |  |  |  |  |  |  |  |
|  |  | green |  | 9/34 | green |  | 28/35 |  |  | green |  | 11/34 |  | green |  | 29/35 |
|  |  | red |  | 24/34 | red |  | 1/35 |  |  | red |  | 23/14 |  | red |  | 6/35 |
|  |  | black |  | 1/34 | black |  | 6/35 |  |  | black |  | 0/14 |  | black |  | 0/35 |
| DF1 | 13°line |  |  |  |  |  |  | PS2 | 14°line |  |  |  |  |  |  |  |
|  |  | green |  | 8/34 | green |  | 22/35 |  |  | green |  | 12/34 |  | green |  | 24/35 |
|  |  | red |  | 20/34 | red |  | 8/35 |  |  | red |  | 18/34 |  | red |  | 8/35 |
|  |  | black |  | 6/34 | black |  | 5/35 |  |  | black |  | 4/34 |  | black |  | 3/35 |
| PA1 | 15°line |  |  |  |  |  |  | DD1 | 16°line |  |  |  |  |  |  |  |
|  |  | green |  | 8/34 | green |  | 25/35 |  |  | green |  | 18/34 |  | green |  | 26/35 |
|  |  | red |  | 15/34 | red |  | 8/35 |  |  | red |  | 16/34 |  | red |  | 9/35 |
|  |  | black |  | 11/34 | black |  | 2/35 |  |  | black |  | 0/34 |  | black |  | 0/35 |
| EF1 | 17°line |  |  |  |  |  |  | LA2 | 18°line |  |  |  |  |  |  |  |
|  |  | green |  | 10/34 | green |  | 28/35 |  |  | green |  | 16/34 |  | green |  | 30/35 |
|  |  | red |  | 17/34 | red |  | 3/35 |  |  | red |  | 16/34 |  | red |  | 2/35 |
|  |  | black |  | 7/34 | black |  | 4/35 |  |  | black |  | 2/34 |  | black |  | 3/35 |
| BO1 | 19°line |  |  |  |  |  |  | CT2 | 20°line |  |  |  |  |  |  |  |
|  |  | green |  | 12/34 | green |  | 27/35 |  |  | green |  | 14/34 |  | green |  | 25/35 |
|  |  | red |  | 16/34 | red |  | 5/35 |  |  | red |  | 18/34 |  | red |  | 7/35 |
|  |  | black |  | 6/34 | black |  | 3/35 |  |  | black |  | 2/34 |  | black |  | 3/35 |
| BB2 | 21°line |  |  |  |  |  |  | LA1 | 22°line |  |  |  |  |  |  |  |
|  |  | green |  | 15/34 | green |  | 25/35 |  |  | green |  | 11/34 |  | green |  | 25/35 |
|  |  | red |  | 18/34 | red |  | 2/35 |  |  | red |  | 14/34 |  | red |  | 4/35 |
|  |  | black |  | 1/34 | black |  | 8/35 |  |  | black |  | 9/34 |  | black |  | 6/34 |
| LM2 | 23°line |  |  |  |  |  |  | PG1 | 24°line |  |  |  |  |  |  |  |
|  |  | green |  | 9/34 | green |  | 25/35 |  |  | green |  | 17/34 |  | green |  | 25/35 |
|  |  | red |  | 14/34 | red |  | 3/35 |  |  | red |  | 17/34 |  | red |  | 10/35 |
|  |  | black |  | 11/34 | black |  | 7/35 |  |  | black |  | 0/34 |  | black |  | 0/35 |
| BF2 | 25°line |  |  |  |  |  |  | HP2 | 26°line |  |  |  |  |  |  |  |
|  |  | green |  | 11/34 | green |  | 25/35 |  |  | green |  | 9/34 |  | green |  | 23/35 |
|  |  | red |  | 23/34 | red |  | 10/35 |  |  | red |  | 19/34 |  | red |  | 5/35 |
|  |  | black |  | 0/34 | black |  | 0/35 |  |  | black |  | 6/34 |  | black |  | 7/35 |
| CC2 | 27°line |  |  |  |  |  |  | DF2 | 28°line |  |  |  |  |  |  |  |
|  |  | green |  | 16/34 | green |  | 21/35 |  |  | green |  | 16/34 |  | green |  | 21/35 |
|  |  | red |  | 13/34 | red |  | 8/35 |  |  | red |  | 12/34 |  | red |  | 8/35 |
|  |  | black |  | 5/34 | black |  | 6/35 |  |  | black |  | 6/34 |  | black |  | 6/35 |
| RG2 | 29°line |  |  |  |  |  |  | CD2 | 30°line |  |  |  |  |  |  |  |
|  |  | green |  | 10/34 | green |  | 26/35 |  |  | green |  | 13/34 |  | green |  | 24/35 |
|  |  | red |  | 16/34 | red |  | 5/35 |  |  | red |  | 21/34 |  | red |  | 11/35 |
|  |  | black |  | 8/34 | black |  | 4/35 |  |  | black |  | 0/34 |  | black |  | 0/35 |
| PG2 | 31°line |  |  |  |  |  |  | FP2 | 32°line |  |  |  |  |  |  |  |
|  |  | green |  | 15/34 | green |  | 23/35 |  |  | green |  | 16/34 |  | green |  | 20/35 |
|  |  | red |  | 11/34 | red |  | 9/35 |  |  | red |  | 10/34 |  | red |  | 8/35 |
|  |  | black |  | 8/34 | black |  | 3/35 |  |  | black |  | 8/34 |  | black |  | 7/35 |

**Supplementary Table 2.** Data from HeatMap. Bacteria are indicated by letters as ID and relative probe number. Dots *per* line of bacteria are presented as ratio amongst green, red and black count (#dot/tot).
